# Influence of extended depth of focus intraocular lenses on visual field sensitivity

**DOI:** 10.1371/journal.pone.0237728

**Published:** 2020-09-14

**Authors:** Makiko Takahashi, Chiemi Yamashiro, Takuya Yoshimoto, Yuka Kobayashi, Fumiaki Higashijima, Masaaki Kobayashi, Makoto Hatano, Manami Ohta, Tomohiko Nagai, Shinichiro Teranishi, Katsuyoshi Suzuki, Ryu Takabatake, Kazuhiro Kimura

**Affiliations:** 1 Takabatake West Eye Clinic, Okayama City, Okayama, Japan; 2 Department of Ophthalmology, Yamaguchi University Graduate School of Medicine, Ube City, Yamaguchi, Japan; National Taiwan University Hospital, TAIWAN

## Abstract

**Purpose:**

To investigate the influence of EDOF IOLs, TECNIS Symfony^®^ (Johnson & Johnson Surgical Vision, Inc.), on visual field sensitivity and to compare the IOLs with other kinds of IOLs.

**Methods:**

The subjects included the normal fellow eyes of patients who underwent the Humphrey Field Analyzer (HFA) 30–2 with Swedish Interactive Threshold Algorithm Fast within 6 months after cataract due to glaucoma or suspected glaucoma. Each parameter of HFA was compared among eyes implanted with TENIS Symfony^®^ (EDOF group), diffractive bifocal IOLs (bifocal group), and monofocal IOLs (monofocal group).

**Results:**

The total of 76 eyes, including 24 eyes in the EDOF group, 26 eyes in the bifocal group, and 26 eyes in the monofocal group, were included in this study. Mean deviation (MD) of HFA was -0.24±0.58 dB in the EDOF group, -1.38±0.58 dB in the bifocal group, and 0.02±0.44 dB in the monofocal group. Foveal threshold (FT) of HFA was 35.8±1.6 dB in the EDOF group, 33.6±1.7 dB in the bifocal group, and 36.6±1.4 dB in the monofocal group. In both MD and FT, there was significant difference between the bifocal group and the others (p<0.001). There was no difference between the EDOF group and the monofocal group. Moreover, there was no significant difference between the three groups about pattern standard deviation (PSD) of HFA.

**Conclusion:**

TECNIS Symfony^®^ may have little influence on visual field sensitivity, whereas diffractive bifocal IOLs decrease visual field sensitivity.

## Introduction

Multifocal intraocular lenses (IOLs) can yield a better quality of life (QOL) and higher satisfaction of patients due to decreasing spectacle dependence rate. On the other hand, diffractive bifocal IOLs also provide good uncorrected visual acuity both in the far and near, although the IOLs contribute to the low contrast sensitivity owing to distributing the incident light to the distance and near images [1–4]. Given the fact, when contrast sensitivity reduction is unacceptable because of some diseases, such as glaucoma and retinal diseases, these kinds of IOLs should be selected more carefully.

Recently, new concept of IOLs, called extended depth of focus IOLs (EDOF IOLs), have been released. The IOLs broaden the range of clear vision due to expanding the range of depth, which provides natural appearance for patients without a decline at middle-distance. TECNIS Symfony^Ⓡ^ (produced from Johnson & Johnson Surgical Vision, Inc.) is one of the EDOF IOLs using a unique technology that enhances the correction of chromatic aberration and that maintains good contrast sensitivity as well as monofocal IOLs [5–7]. It is possible that EDOF IOLs become indicated for patients who have not been a candidate for diffractive bifocal IOLs. Several studies have recently addressed that multifocal IOLs decrease mean deviation (MD) of VF test using (Humphrey field analyzer, Carl Zeiss Meditec, Inc.) compared to diffractive bifocal IOLs, monofocal IOLs, or phakic eyes [8, 9]. A report shows a correlation between contrast sensitivity and visual field (VF) sensitivity [10]. However, to our knowledge, there were no reports on the relationship between EDOF IOLs, which may not decrease contrast sensitivity, and VF sensitivity. This study investigated the influence of TECNIS Symfony^Ⓡ^, one of the EDOF IOLs, on VF sensitivity.

## Materials and methods

### Patients

This study was approved by the Institutional Review Board (IRB) of Yamaguchi University and examined retrospectively. We received ethical approval prior to accessing and analyzing the data. The subjects included the fellow eyes of patients who underwent a VF test for glaucoma or suspected glaucoma within 6 months after uniocular cataract surgery from July 2013 until March 2019 at Takabatake West Eye Clinic and Yamaguchi University. There is no glaucomatous or other abnormal finding of disc shape and retinal nerve fiber layer in these fellow eyes under the fundus photography and spectral-domain optical coherence tomography (3D OCT-2000, Topcon). There is also no glaucomatous or other abnormal finding of VF under VF test using HFA. Exclusion criteria were defined as follows: (1) logMAR corrected visual acuity <0.0 (naked eyes corrected logMAR visual acuity for diffractive bifocal IOLs <0.0) (2) Adults aged ≥70 years (3) ocular axial length of ≥26 mm (4) ≥ 15% in any reliable indicators of the VF test (fixation loss, false positive, and false negative), and (5) ocular disease that may affect the VF. A retrospective study was performed on 76 eyes with respect to the following parameters: sex, age, best corrected visual acuity (logMAR), spherical equivalent, and ocular axial length.

The subjects were allocated into three groups by the kind of IOLs. We defined eyes implanted with TECNIS Symfony^Ⓡ^ ZXR00V as the EDOF group, diffractive bifocal IOLs, such as TECNIS^Ⓡ^ Multifocal including ZLB00 and ZMB00 (Johnson & Johnson Surgical Vision, Inc.), as the bifocal group, and monofocal IOLs, such as TECNIS^Ⓡ^ZCB00 (Johnson & Johnson Surgical Vision, Inc.) or Nex-Acri^Ⓡ^ N4-18YG (NIDEK, CO., LTD.) as the monofocal group.

### Visual field test

**Fig 1:**
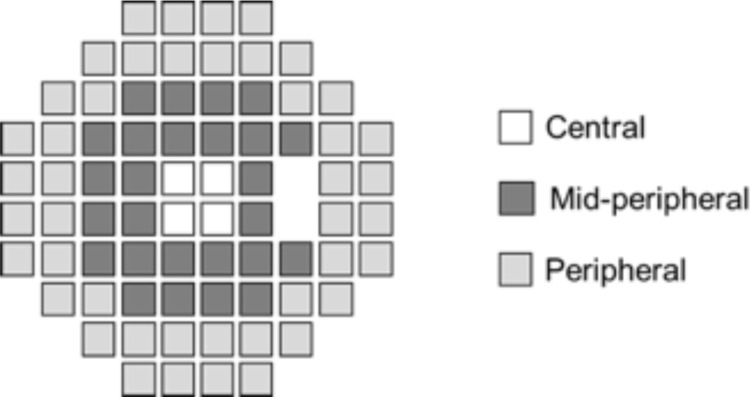
Three visual fields sectors. The TD was divided into three areas (central, midperipheral, and peripheral) of the HFA.

HFA30-2 Swedish Interactive Threshold Algorithm Fast was used for measuring the VF. Foveal threshold (FT), MD, total deviation (TD), and pattern standard deviation (PSD) were evaluated. TD was divided into three areas (central, midperipheral, and peripheral areas) without Mariotte blind spot (Fig 1) based on the important location of the VF for the quality of vision such as reading and mobility [11], which were compared by average value for each area. Then, we compared the difference in these parameters among EDOF group, bifocal group and monofocal group. All patients underwent VF testing with the best corrected near vision using add-on lens subjectively if needed. No patients in the bifocal group required add-on lens for correcting the focus under VF test. All patients underwent VF test using HFA at the first time or the second time. Reliable results of VF tests according to the above exclusion criteria were applied to data analysis.

### Statistical analysis

As a statistical analysis method, *x*^2^ test was used for sex. For the other indicators, a one-way analysis of variance (ANOVA) was used in this study. When ANOVA showed a significant difference, Student’s t-test was used for multiple comparisons. The significance level was set at p<0.05. Results are expressed as mean ± standard deviation (SD).

## Results

Of the 76 eyes in 76 patients, 24 eyes were in the EDOF group, 26 eyes were in the bifocal group, and 26 eyes were in the monofocal group. The patient background of each group was shown in Table 1. The spherical equivalent of the monofocal group was significantly lower compared to that of the EDOF group (p = 0.002) and the bifocal group (p<0.001) respectively. The spherical equivalent of the EDOF group was significantly lower compared to that of the bifocal group (p = 0.007). The other indicators had no significant differences. The bifocal group included 21 eyes implanted with TECNIS^®^ Multifocal ZLB00, and 5 eyes implanted with TECNIS^®^ Multifocal ZMB00. The monofocal group included 20 eyes implanted with TECNIS^®^ ZCB00, and 6 eyes implanted with Nex-Acri^®^ N4-18YG.

**Table 1:** Demographic data of IOL implanted eyes.

	EDOF (n = 24)	Bifocal (n = 26)	Monofocal (n = 26)	P Value
Sex (male/female)	13/11	12/14	9/17	0.375 ^1)^
Age (year)	62.6±7.8	62.6±7.3	65.7±6.1	0.208 ^2)^
BCVA (logMAR)	-0.16±0.05	-0.16±0.04	-0.14±0.05	0.340 ^2)^
SE (D)	-0.32±0.56	0.02±0.25	-1.18±1.20	<0.001 ^2)^
Axial length (mm)	24.0±1.3	24.7±1.2	24.3±1.4	0.114 ^2)^

BCVA: Best-Corrected Visual Acuity; SE: Spherical Equivalent.

^1)^ Chi-square test;

^2)^ ANOVA test.

FT value of each group was 35.8±1.6 dB in the EDOF group, 33.6±1.7 dB in the bifocal group, and 36.6±1.4 dB in the monofocal group (Table 2). MD value of each group was -0.24±0.58 dB in the EDOF group, -1.38±0.58 dB in the bifocal group, and +0.02±0.44 dB in the monofocal group. As regards both MD and FT values, these of the bifocal group were significantly lower than the others (p<0.001), and there was no significant difference between the EDOF group and the monofocal group. As for PSD values, there was no significant difference among the three groups.

**Table 2:** Comparison on indices of visual field in IOL implanted eyes.

	EDOF (n = 24)	Bifocal (n = 26)	Monofocal (n = 26)	P Value*
MD (dB)	-0.24±0.58	-1.38±0.58	0.02±0.44	<0.001
PSD (dB)	1.60±0.34	1.51±0.19	1.54±0.28	0.463
FT (dB)	35.8±1.6	33.6±1.7	36.6±1.4	<0.001

MD: Mean Deviation; PSD: Pattern Standard Deviation; FT: Foveal Threshold.

*Student’s-t test.

The average value of TD at each area, including central, midperipheral, and peripheral areas, was shown in Fig 1. The average TD value at each area in the bifocal group was significantly lower than that of the EDOF group and the monofocal group (p<0.01) (Table 3). The average TD value of the EDOF group was significantly lower than that of the monofocal group at the midperipheral area (p<0.05), whereas no difference was detected at the central or peripheral area.

**Table 3:** Comparison on total deviation at three areas of visual field in IOL implanted eyes.

	EDOF (n = 24)	Bifocal (n = 26)	Monofocal (n = 26)	P Value*
Central (dB)	0.38±0.76	-1.17±0.83	0.47±0.84	<0.001
Midperipheral (dB)	-0.29±0.62	-1.58±0.57	0.05±0.55	<0.001
Peripheral (dB)	-0.54±0.73	-1.17±0.84	-0.17±0.75	<0.001

*Student’s-t test.

## Discussion

With regard to the influence of multifocal IOLs on VF sensitivity, Aychoua et al have reported that in the multifocal IOLs (TECNIS^®^ Multifocal ZM900; AMO, AT LISA^®^ 809M; CarlZeiss Meditec), the MD value of HFA was about 2 dB lower than the monofocal IOLs [9]. Farid et al also reported that in the multifocal IOLs (TECNIS^®^ Multifocal ZMB00, ZMA00; AMO, ReSTOR^®^ SN6AD1; Alcon), the MD value of HFA was about 2 dB lower [8]. In this study, eyes implanted with diffractive bifocal IOLs did not only reduce the FT value by 3.0 dB in HFA but also the MD value by 1.4 dB, compared to eyes implanted with monofocal IOLs. Furthermore, eyes implanted with diffractive bifocal IOLs decreased the TD value among them, but the difference of PSD value was not detected. These results suggest that diffractive bifocal IOLs reduce the sensitivity of the full VF. In contrast, TECNIS Symfony^®^, one of the EDOF IOLs, was even to monofocal IOLs in terms of both MD and FT values. Diffractive bifocal IOLs tend to suppress the contrast sensitivity due to the optical characteristics dividing the focal point between far and near [1–4, 6]. Whereas, TECNIS Symfony^®^ was able to suppress the decrease in the contrast sensitivity by improving the design fo diffraction [5, 6]. This can explain that the difference in contrast sensitivity, owing to the difference in the structure of the IOLs, was reflected in the VF sensitivity.

Various potential contributing factors could have an influence on VF sensitivity of multifocal IOLs on VF sensitivity. Patient age has an effect on visual function in the patients with multifocal IOLs compared to the patients with monofocal IOLs [12]. In this study, we focused on patients under 70 years of age. Near visual acuity or contrast sensitivity of the eyes implanted with multifocal IOLs are also susceptible to even mild posterior capsule opacity [13]. Therefore, we analyzed the VF within 6 months after cataract surgery to avoid the influence of cataract as much as possible. It was reported that the best visual function requires a brain acclimatization period due to the special optical structure of diffractive bifocal IOL [14, 15]. Farid et al reported that there was no difference between MD value of the VF test within 6 months or after cataract surgery [8]. Given the fact, we considered that the timing of examination had little effect on the MD value. The ocular axial length is also one of the factors affecting the VF sensitivity [16–18]. Rudnicka et al reported that, in a myopic eye whose ocular axial length was 26 mm or more, as the axial length is longer every 1 mm, there was a 0.8 dB decrease in the MD value of HFA [18]. We, therefore, excluded high myopic eyes with 26 mm or more axial length in this study. In addition, this study was conducted with sound eyes without any ocular diseases that may affect their VF. There were no differences among the three groups about visual acuity or ocular axial length.

It was previously reported that diffractive bifocal IOLs decreased the MD value [8, 9]. In this study, we showed the MD value decreased in patients with diffractive bifocal IOLs but the FT value and all VF also decreased significantly compared to the monofocal IOLs. VF sensitivity is correlated with the National Eye Institute 25-Item Visual Function Questionnaire (NVI-VFQ25), which is the index of vision-related QOL [19]. Especially, there is a strong correlation between NVI-VFQ25 and the desensitization of the central cluster [20–22]. Patients with diffractive bifocal IOLs may decrease their visual function and compromise their QOL compared to patients who have low VF sensitivity by progressing glaucoma or retinal diseases [23, 24]. Implantation with diffractive bifocal IOLs would cause further deterioration of contrast sensitivity to patients who underwent laser in situ keratomileuses (LASIK) [25] because of decreasing contrast sensitivity after LASIK [26]. The result of our study suggests that we must be careful in implanting diffractive bifocal IOLs to such cases that may reduce the contrast sensitivity radically.

We showed that diffractive bifocal IOLs promoted the decrease in TD among central, midperipheral, and peripheral areas compared to monofocal IOLs. Van der Mooren et al have reported that retinal straylight is associated with the elevation in luminance detection thresholds and reductions in contrast sensitivity [27]. Retinal straylight of diffractive bifocal IOLs is stronger than that of monofocal IOLs [28]. These results suggest that diffractive bifocal IOLs may reduce the TD value at all areas mediated by straylight. TD values of TECNIS Symfony^®^ at central and peripheral areas were almost equivalent to those of monofocal IOLs. These results demonstrate that TECNIS Symfony^®^ has little effect on the central VF that has been strongly associated with QOL. In contrast, the TD value of TECNIS Symfony^®^ at midperipheral area was significantly lower than that of monofocal IOLs. The optical quality of TECNIS Symfony^®^ is almost similar to that of monofocal IOLs [29]. However, TECNIS Symfony^®^ has an elongated focus although monofocal IOLs have a fixed focus for one distance. These results suggest that straylight may have an influence on the peripheral area exhibiting reduced retinal sensitivity at EDOF IOLs.

TECNIS Symfony^®^ uses the company`s own Echellet diffraction technique to extend the depth of focus, and correct chromatic aberration with achromatic technology. Therefore, TECNIS Symfony^®^ maintains high contrast sensitivity like monofocal IOLs in spite of having a wide clear vision region [5, 6]. A report has addressed the correlation between contrast sensitivity and VF sensitivity [10]. This study also showed that both MD and FT values and the central visual sensitivity of eyes implanted with TECNIS Symfony^®^ were better than those of eyes implanted with diffractive bifocal IOLs. They were almost equivalent to those of eyes implanted with monofocal IOLs [29]. In this context, we considered that TECNIS Symfony^®^ is the IOLs that have little influence on VF sensitivity and would be adequate to be implanted for patients who need to have VF tests regularly even after cataract surgery. It was reported that eyes implanted with TECNIS Symfony^®^ had an uneventful course after LASIK [30]. Based on these findings, it is conceivable that TECNIS Symfony^®^ has a wide indication.

In conclusion, our study showed that TECNIS Symfony^®^, one of the EDOF IOLs, has little influence on VF sensitivity and may be a candidate for patients who have not been encouraged to implant the diffractive bifocal IOLs.

## Supporting information

S1 Data (XLSX)
